# Barriers and enablers to and strategies for promoting domestic plasma donation throughout the world: Overarching protocol for three systematic reviews

**DOI:** 10.1371/journal.pone.0296104

**Published:** 2023-12-21

**Authors:** Cole Etherington, Amelia Palumbo, Kelly Holloway, Samantha Meyer, Maximillian Labrecque, Kyle Rubini, Risa Shorr, Vivian Welch, Emily Gibson, Terrie Foster, Jennie Haw, Elisabeth Vesnaver, Manavi T. Maharshi, Sheila F. O’Brien, Paul MacPherson, Joyce Dogba, Tony Steed, Mindy Goldman, Justin Presseau

**Affiliations:** 1 Clinical Epidemiology Program, Ottawa Hospital Research Institute, Ottawa, Ontario, Canada; 2 Canadian Blood Services, Ottawa, Ontario, Canada; 3 School of Public Health Sciences, University of Waterloo, Waterloo, Ontario, Canada; 4 Lived Experience Partner, Calgary, Alberta, Canada; 5 Lived Experience Partner, London, Ontario, Canada; 6 Learning Services, The Ottawa Hospital, Ottawa, Ontario, Canada; 7 School of Epidemiology and Public Health, University of Ottawa, Ottawa, Ontario, Canada; 8 Campbell Collaboration, Ottawa, Ontario, Canada; 9 Division of Infectious Diseases, The Ottawa Hospital, Ottawa, Ontario, Canada; 10 Faculty of Medicine, Université Laval, Québec City, Québec, Canada; Seoul St. Mary’s Hospital, College of Medicine, The Catholic University of Korea, REPUBLIC OF KOREA

## Abstract

**Introduction:**

The growing demand for plasma protein products has caused concern in many countries who largely rely on importing plasma products produced from plasma collected in the United States and Europe. Optimizing recruitment and retention of a diversity of plasma donors is therefore important for supporting national donation systems that can reliably meet the most critical needs of health services. This series of three systematic reviews aims to synthesize the known barriers and enablers to source plasma donation from the qualitative and survey-based literature and identify which strategies that have shown to be effective in promoting increased intention to, and actual donation of, source plasma.

**Methods and analysis:**

Primary studies involving source or convalescent plasma donation via plasmapheresis will be included. The search strategy will capture all potentially relevant studies to each of the three reviews, creating a database of plasma donation literature. Study designs will be subsequently identified in the screening process to facilitate analysis according to the unique inclusion criteria of each review (i.e., qualitative, survey, and experimental designs). The search will be conducted in the electronic databases SCOPUS, MEDLINE, EMBASE, PsycINFO, and CINAHL without date or language restrictions. Studies will be screened, and data will be extracted, in duplicate by two independent reviewers with disagreements resolved through consensus. Reviews 1 and 2 will draw on the Theoretical Domains Framework and Intersectionality, while Review 3 will be informed by Behaviour Change Intervention Ontologies. Directed content analysis and framework analysis (Review 1), and descriptive and inferential syntheses (Reviews 2 and 3), will be used, including meta-analyses if appropriate.

**Discussion:**

This series of related reviews will serve to provide a foundation of what is known from the published literature about barriers and enablers to, and strategies for promoting, plasma donation worldwide.

## Introduction

The demand for plasma protein products, such as immunoglobulin (Ig), is steadily growing across the globe with the emergence of new products and applications, diagnostic advances, and increased access to medical care. [1,2] Plasma, a protein-rich liquid that makes up ~55% of the overall blood volume, is the foundation of a well-functioning immune system and responsible for moving all parts of blood through the body. [3] The proteins in plasma are used to develop treatment for immune deficiencies, bleeding disorders, kidney & liver diseases, burns, surgery and cancer. [3] Unlike whole blood donation, plasma donation can be done as often as every one to two weeks in some countries, [4] but involves a greater time commitment (approximately 45 to 60 minutes vs. 15 minutes), a greater volume of fluid being collected, and reinfusion of blood cells and platelets.

The global market for Ig therapies, one of the most common plasma-derived products, is experiencing annual growth between 6% and 8%. [5] The World Health Organization (WHO) has emphasized the importance of countries achieving self-sufficiency through voluntary (non-remunerated) plasma donations as a key national objective to ensure a secure and adequate supply for their respective populations [6]. However, there exists a notable geographical disparity in plasma supply, with only a few countries collecting enough to meet their population’s needs. Many nations depend on the United States (US) and European countries that permit donor remuneration (Germany, Austria, Czech Republic, Hungary) [1,7,8]. Approximately 65% of the world’s plasma supply comes from the US, while over 55% of European plasma is contributed by Germany, Austria, the Czech Republic, and Hungary [7,8]. This reliance on a limited number of countries poses a potential challenge to plasma supply, raising the risk of shortages in case of market disruptions. Notably, Ig therapies are frequently affected by shortages in Europe, and shortages have recently been experienced in Canada. Supply interruptions remain a serious concern due to increasing costs driven by international competition, especially as the demand for Ig rises in emerging markets. In addition, the overall decline in plasma donations due to the COVID-19 pandemic [9] and the potential for future supply chain disruptions during similar international events suggest a need for improve domestic plasma collection strategies [7,10]. This could also reduce the carbon footprint that likely results from of shipping plasma internationally.

In light of the increasing global reliance on the EU and US for source plasma, countries such as Canada have developed plasma sustainability programs to boost domestic plasma collection and ensure continuity of supply [7]. These programs include domestic fractionation facilities, increased availability of plasma donation sites, and improved recruitment of first-time donors directly into plasma donation programs [11], as opposed to previous models focused on converting whole blood donors to plasma donation [12,13]. Several new plasma-only donation centres have been opened across the country and donation eligibility criteria have evolved to include a diversity of donors [14]. Although steps such as these are necessary for increasing the self-sufficiency of plasma donation systems, they are not sufficient given the wide range of known barriers and enablers to donating plasma (e.g., awareness, time, accessibility) [15–21]. At the same time, it is also unclear how barriers and enablers, along with donation promotion strategies, may vary across cultural contexts. For example, there may be important regional differences to consider as regulatory frameworks governing plasma donation are country specific [4,7]. Perceptions, beliefs, and norms associated with plasma donation may also differ across different contexts, along with the potential influence of economic conditions and disparities with regard to donor remuneration [4,7,22]. A systematic assessment of these potential differences can inform context-specific adaptations of future interventions.

An additional challenge facing plasma donation systems involves the multifaceted nature of barriers and enablers within diverse communities. Experiences of exclusion and negative encounters within the donation system among various marginalized communities, including racialized, indigenous, and Two-Spirit, lesbian, gay, bisexual, transgender, and queer (2SLGBTQ+) groups [16,23–27], can significantly impact both the recruitment and retention of donors. Recent qualitative work with gay, bisexual and other men who have sex with men, for example, suggests institutionalized discrimination and stigma are key concerns regarding donation [17]. Thus, it is imperative to ensure developed interventions do not generate inequities or perpetuate additional bias and discrimination [28]. Understanding how barriers and enablers to donation, as well as donation strategies, may vary within and between groups is an important first step in this regard.

Ensuring national donation systems can reliably meet the most critical needs of health services, while also upholding principles of equity, diversity and inclusion, remains a considerable public health concern. A recent rapid review on plasma donation has highlighted the need for a comprehensive, systematic, and multi-level analysis of the literature to better inform future plasma collection strategies [21]. Broadening the focus of this review and performing a sequence of theory-driven systematic analyses will enrich our understanding of both the behavioural and contextual aspects of plasma donation, with direct implications for intervention development.

### Objectives

The overarching goal of this work is to conduct a theory-informed synthesis of the qualitative, survey, and intervention literature on factors associated with plasma donation motivation, initiation, and maintenance. This will be accomplished through three separate systematic reviews that each focus on a particular research design. Each design lends itself to the study of a different aspect of plasma donation (i.e., qualitative for barriers and enablers; surveys for associated factors; experimental for testing donation strategies), and therefore, different types of analyses.

#### Review 1

Meta-synthesis of barriers and enablers to plasma donation across the qualitative literature: This meta-synthesis aims to (1) synthesize barriers and enablers to source plasma donation using the Theoretical Domains Framework [29,30]; and (2) classify identified barriers and enablers according to a multi-level (i.e., individual, interpersonal, structural) intersectional framework [31–33].

#### Review 2

Systematic review of factors associated with plasma donation identified in the survey literature: This systematic review will identify, synthesize, and analyze the relationship between factors associated with intention to and actual donation of plasma within the quantitative survey literature. The Theoretical Domains Framework [29,30] will be used as a synthesising framework. Variation within and across intersecting categories of participants will be conducted along with an assessment of the relative effect of individual vs. structural-level factors (if possible).

#### Review 3

Systematic review of trials and quasi-experiments testing the effectiveness of interventions to promote plasma donation: This systematic review will identify studies testing the effectiveness of interventions to promote plasma donation and assess their effects on intention to and actual donation of plasma. This review will also classify the active components in existing plasma donation interventions (and control conditions) using the Behaviour Change Technique Taxonomy version 1, and modes, setting, and source of intervention delivery will also be classified according to their respective behaviour change intervention ontologies.

## Methods

Each systematic review will be conducted in accordance with the Preferred Reporting Items for Systematic Reviews and Meta-Analyses (PRISMA) 2020 guideline (S1 Appendix) [34]. A pre-print for this protocol has been registered on Open Science Framework (https://psyarxiv.com/seprt/).

### Analytical frameworks

#### Reviews 1 and 2

Theoretical Domains Framework (TDF): The TDF was developed and validated to identify potential modifiable factors that can be targeted by behaviour change interventions [29]. By synthesizing 33 psychological theories of behaviour into 14 domains [29], the TDF is a useful framework through which to identify facilitators of and barriers to change [30]. It has been widely applied across healthcare settings, and most recently, to understand barriers and enablers to implementing new plasma donation criteria in Canada [16]. While the TDF helps to categorize cognitive, affective, social and environmental determinants of individual behaviour [30], there is also an opportunity to better understand the interplay of social identity and structural context with these determinants through incorporation of an intersectional lens [28,35,36].

Intersectionality: Intersectionality was originally developed by Black feminist scholars and activists [32], recognizing that individuals’ lived experiences are simultaneously shaped by many social identities (e.g., indigeneity, race, gender, sexual orientation) and their dynamic interaction with systems of power and oppression (e.g., colonialism, racism, sexism, transphobia, homophobia) [37]. In drawing attention to the complexity and multidimensionality of individuals’ lives within broader social and structural contexts, intersectionality also provides a way to categorize experiences into three levels of influence: macro, meso, and micro [33,38]. The macro, or structural level, involves “policies, practices, norms, power structures, and dynamics” [39] create social hierarchies, impose constraints, and restrict opportunities for marginalized individuals and groups [33,38,39]. The meso, or interpersonal level, involves interactions with social actors within groups, communities, organizations, and institutions that perpetuate bias and discrimination [33,38,39]. Finally, the micro (i.e., intrapersonal or individual) level includes individuals’ social location and identities (e.g., age, gender, sex, indigeneity, race, ethnicity, sexual orientation, housing situation, work history, family status) [33,38,39]. Moreover, it is where experiences with the macro and meso levels are felt–that is, it is the social psychological context for individuals who experience bias, stigma, and discrimination based on their social identities [39,40]. This may include internalized stigma, a sense of powerlessness, and cumulative stress [39,41–44].

#### Review 3

Behaviour Change Intervention Ontologies: An ontology is a structure for organizing and representing information. Individual units within are called “entities” and the relationships between these entities are defined in a hierarchical system. Ontologies are similar to taxonomies, but a key difference is their machine-readability, which can make organization and analyses of large datasets labelled with ontological labels much easier to automate. Several ontologies have been developed by a group of behaviour change experts as part of the Human Behaviour-Change Project under the overarching Behaviour Change Intervention Ontology [45]. The ontologies are developed through such methods as review and annotation of behaviour change interventions in the existing literature and expert stakeholder review. Broad use of the ontologies can enable better classification, synthesis, analysis, and replication of behaviour change interventions across their varying components. At the time of writing, four ontologies have been developed that represent intervention content (Behaviour Change Techniques Ontology), delivery (Mode of Delivery Ontology, Intervention Source Ontology), and context (Intervention Setting Ontology).

The Behaviour Change Techniques Ontology builds on the Behaviour Change Techniques Taxonomy version 1 and seeks to enable consistent and accurate descriptions of the active components in interventions to reduce confusion caused by overlapping or inconsistent definitions of behaviour change intervention content [46]. A behaviour change technique (BCT) is an “observable, replicable, and irreducible component of an intervention designed to alter or redirect causal processes that regulate behaviour” [46] and the Behaviour Change Techniques Ontology provides a common language for labelling these components. The Behaviour Change Techniques Ontology organizes 259 BCTs into 20 higher-level groups.

Intervention delivery can also vary widely, for example by mode, schedule, style, or source of delivery, and it has been suggested that the effectiveness of an intervention can be influenced by its delivery mechanisms [45]. The Mode of Delivery Ontology was developed to assist in labelling the “physical or informational medium through which a behaviour change intervention is provided” and organizes 65 modes of delivery into 15 higher-level groups [47]. The Intervention Source Ontology^48^ was developed to assist in labelling the person, population, or organization providing the intervention and consists of 140 entities [48].

The context of a behaviour change intervention, including the setting in which it is provided and the population to whom it is provided, can also vary greatly. Despite the recommendations in standardized reporting guidelines such as the CONsolidated Standards of Reporting Trials statement (CONSORT) [49] and the Template for Intervention Description and Replication checklist (TIDieR) [50] including reporting intervention setting, these guidelines do not offer a method to systematically classify them. The Intervention Setting Ontology organizes 72 entities into 2 higher-level groups (i.e., Physical Setting and Social Setting) [48].

As new ontologies from the Human Behaviour-Change Project are released, we will consider applying them to our review if relevant.

### Information sources and search strategy

The search strategy will be designed to capture all potentially relevant studies to each of the three reviews. The reviews focused on qualitative (Review 1) and survey-based (Review 2) literatures will provide a database of plasma donation literature relevant to factors that are linked to greater or less motivation and actional donation (i.e., barriers and enablers affecting capability, opportunity, and/or motivation). The review focused on rigorous tests of plasma donation promoting strategies (Review 3) will further contribute to a database of approaches that have been used and shown to be effective or not in promoting motivation to donate and actual donation. The resultant literature will be available through each systematic review when published as well as linked in the reviews’ open science framework page. Study designs will be subsequently identified in the screening process to facilitate analysis according to the unique inclusion criteria of each review (i.e., qualitative, survey, and experimental designs).

The search strategy will be developed with an experienced academic librarian in collaboration with the research team (S2 Appendix), then reviewed by a second information specialist as per the Peer Review of Electronic Search Strategies (PRESS) guidelines [51]. The search will be conducted in the electronic databases SCOPUS, MEDLINE, EMBASE, PsycINFO, and CINAHL. We initially planned to include Google Scholar but elected to remove it after preliminary searchers were run given the lack of unique relevant references captured. The reference lists of all included studies will also be screened to identify any additional relevant studies. Date and language restrictions will not be imposed; however, only studies published in English or French will be included in the reviews, with a list of potentially relevant studies published in other languages published as an appendix.

### Eligibility criteria

#### Inclusion criteria

Population: Studies involving source or convalescent plasma donors (first-time, converted from whole blood, or repeat), will be eligible for inclusion. Donation can be remunerated or non-remunerated. If a study involves both plasma and blood donors, data on plasma donors must be reported separately for the study to be included.

Phenomena of interest: views/perspectives of individuals eligible to donate plasma and/or barriers or enablers to donating plasma (Review 1); modifiable factors associated with intention to and/or actual donation of plasma (Review 2); effectiveness of interventions promoting plasma donation (Review 3).

Study designs: Peer-reviewed original study: primary qualitative data generated through interviews, focus groups and other qualitative methods (Review 1); quantitative cross-sectional or longitudinal survey research testing an association between a construct and intention/behaviour (Review 2); interventional designs including but not limited to randomized controlled trials (RCTs), quasi-RCTs, cluster RCTs, and controlled before-and-after studies (Review 3).

Language: full-text published in English or French.

#### Exclusion criteria

Commentaries, conference abstracts, dissertations, and grey literature sources will be excluded along with any study focusing only on blood donors or not reporting data on plasma donors separately from blood donors (for studies including both).

### Study selection and data extraction

Search results will be uploaded into DistillerSR (Evidence Partners, Ottawa, Canada), an online systematic review software. A screening form will be developed by the author team a priori and piloted with a subset of 10 references to establish inter-rater reliability. Two reviewers will independently screen studies by title and abstract, with studies identified as meeting or potentially meeting inclusion criteria proceeding to full-text review. Full texts will be screened independently and in duplicate by the two reviewers, with disagreements resolved through discussion or by consulting with a third reviewer if needed. Data will be extracted by the reviewers independently using a data collection form developed by the research team. The form will capture information such as publication information (e.g., first author, year of publication, country of data collection); study aim, design, and setting; sample size; participant demographic characteristics, methods, outcomes, and results related to each of the review objectives. If any data are missing, we will attempt to contact study authors to request them.

### Quality appraisal and risk of bias assessment

Study quality and risk of bias will be reported descriptively using the following tools, as appropriate.

The CASP Qualitative Checklist [52] will be used to assess the quality of included qualitative studies (Review 1), as recommended by Cochrane [53]. The CASP tool involves ten questions to assist with evaluation of a study’s rigour, credibility, and relevance [52].

The Newcastle-Ottawa Scale (NOS) will be used to assess risk of bias for included survey studies (Review 2). The NOS examines risk of bias across three domains: selection of study groups, comparability of groups, and ascertainment of exposure and outcomes. It has been adapted for both cross-sectional and cohort studies [54].

For studies of interventions, the Cochrane Risk of Bias 2 Tool (RoB2) [55] and the Risk of Bias in Non-randomized Studies of Interventions (ROBINS-I) tool [56] will be used, as appropriate. RoB2 assesses bias in five domains (bias arising from the randomization process, bias due to deviations from intended interventions, bias due to missing outcome data, bias in measurement of the outcome, bias in selection of the reported result), leading to an overall risk of bias judgement [55]. The ROBINS-I tool assesses the following domains: bias due to confounding, selection bias, bias in measurement classification of interventions, bias due to deviations from intended interventions, bias due to missing data, bias in measurement of outcomes, bias in selection of the reported result, and overall bias [56]. Furthermore, we will assess heterogeneity using Chi^2^ and I^2^ tests and funnel plots to explore small study or publication biases [57].

### Planned synthesis of results for Review 1

The first step of data synthesis will involve directed content analysis [58] using the TDF as a coding framework. After data are categorized according to the TDF domains, barriers and enablers will be summarized thematically [59]. An additional framework analysis [60] will be applied to summarize and map barriers and enablers across macro, meso, and micro levels of influence. Data coding will be conducted in NVivo using an iteratively developed coding manual.

#### TDF data coding

Extracted data (i.e., the results reported by the included studies) will be coded into relevant TDF domains in duplicate by two independent reviewers. Disagreements will be resolved through consensus discussion between the two reviewers or involvement of a third reviewer as needed. Data items deemed relevant to multiple domains will be coded into each domain separately, and decision rules will be documented in the coding manual. While the TDF synthesizes over 30 behaviour change theories, some themes and measures may not readily map to the TDF and thus we will also conduct an inductive synthesis of non-TDF factors. Potential differences in barriers and enablers according to donor remuneration status and country will also be explored.

#### Intersectional, multi-level analysis

Intersecting social categories of participants across studies will be summarized descriptively. Differences in barriers and enablers across participant identities will be explored. Barriers and enablers will also be deductively coded into the categories of macro, meso, and micro levels of influence. Coding will be conducted by one reviewer with expertise in intersectional, multi-level analysis (CE) and verified by a second reviewer. Discrepancies will be resolved through consensus or consultation with a third reviewer if necessary.

### Planned synthesis of results for Review 2

We will use the coding manual from Review 1 to code constructs in survey studies which will include the list of theoretical constructs included in each TDF domain (24,25) and the macro, meso, and micro levels of influence (28, 33, 34). These will serve to provide consistent labels for the constructs measured across each survey study.

#### Descriptive synthesis

We will extract and summarize study characteristics (author, year, country, theory used [if any], sample size, design [cross-sectional, longitudinal] and participant demographic data from each study (e.g., age, gender, sex, race, ethnicity, sexual orientation), and mean scores on TDF-coded constructs within each survey, as well as the two outcomes of interest (intention to donate and donation behaviour). We will also summarize the extent of measures of donation behaviour based on self-report vs observed. For studies with more than one time point of measurement that include more than one measure of the same construct, we will preference the scores and associations on the earliest measure of the predictors and latest measure of the outcomes (intention and behaviour). Where available we will extract data on psychometrics (eg. Cronbach’s alpha) for each construct in each survey and describe the mean and range of such indices across studies. We will also extract and synthesize the bivariate association between each coded TDF construct and intention and/or behaviour, where available, again preferencing the earliest measurement point of the TDF construct and the latest measurement point of intention/behaviour available. Where available, we will also extract the overall explained variance (R^2^) in studies where a multivariate model is tested to account for variance in intention or behaviour. Variation within and across intersecting categories of participants will be conducted along with an assessment of the relative effect of individual vs. structural-level factors (if possible). A subgroup analysis by country of data collection (Organization for Economic Cooperation and Development [OECD] member vs. not), type of plasma collected (source vs. convalescent), and year of publication will also be conducted.

#### Meta-analysis

We will use Comprehensive Meta-Analysis using a random effects model to synthesize bivariate correlations between TDF-coded constructs present in at least 2 studies, with intention and behaviour. Effect sizes will be described in accordance with Cohen’s recommendations (r = 0.1 small effect size, r = 0.30 medium effect size, and r = 0.50 large effect size). Analysis of heterogeneity will be conducted using Cochrane’s Q and I^2^ statistics. Where correlations are only presented for sub-groups, we will compute a frequency-weighted mean correlation.

### Planned synthesis of results for Review 3

We plan to conduct a meta-analysis of the effect of included interventions on intention/willingness to donate and on donation behaviour as well as a meta-regression of the relative effects of BCTs, modes and sources of delivery, and settings if the sample size of included studies allows.

#### Coding

Intervention content, delivery, and context will be coded based on the BCTTv1, Mode of Delivery Ontology, Intervention Source Ontology, and Intervention Setting Ontology. Two authors with experience in applying the BCTTv1 will first practice by coding a set of papers that are not included in the review and develop a coding manual, then independently code each of the included studies, iteratively refining the coding manual. Consensus meetings will be regularly conducted to identify and resolve coding discrepancies (exact interval to be determined based on number of included papers). In line with a similar review [61], inter-rater agreement will be calculated using Cohen’s Kappa based on the BCTs identified by coders as present rather than absent to reduce the risk of overinflating the coefficient value.

#### Descriptive synthesis

All eligible studies will be included in the descriptive synthesis. Interventions will be described according to their methods (year, design, duration, follow-up, theory used, outcome measure), participants (e.g., country, sample size, sex, gender, race, ethnicity, age), and intervention characteristics (quantity and description of BCTs coded in the intervention and control arms, mode, source, and setting of delivery).

#### Inferential synthesis

We will use a random-effects model using Comprehensive Meta-Analysis software [62], focused on randomized and cluster-randomized trials. We will conduct a pooled analysis of the effect of plasma donation promoting interventions on two outcomes: intention/willingness to donate plasma, and actual plasma donation. When studies have multiple intervention arms, we will combine active intervention arms to form a pairwise comparison. For dichotomous outcomes (e.g., yes/no donation) we will present findings using a risk ratio and 95% confidence interval (CI). For continuous outcomes (e.g., donation frequency), we will use a standardized mean difference (SMD) and 95% CI. Subjectively and objectively measured outcomes will both be extracted and pooled separately; if a study reports both, objectively measured outcomes will have preference for inclusion in the meta-analysis.

We will conduct a meta-regression using a random-effects model to identify the effectiveness of interventions including a singular BCT (univariate analysis). Separate analyses will be conducted for each of the primary outcomes (i.e., willingness/intention to donate and actual donation behaviour). Any BCT that is coded as present in at least three studies will be eligible for inclusion in univariate meta-regression analyses [63]. We will also conduct univariate analyses to test if commonly co-occurring combinations of BCTs are related to the intervention effect size. Any variables that are statistically significant (*p* < .05) will be included in a multivariate meta-regression analysis. Wherever possible, a similar protocol will be followed separately for modes of delivery, sources of delivery, and settings of delivery.

#### Subgroup analyses

One set of subgroup analyses will be undertaken to calculate the effect of interventions in locations where donations are remunerated and the effect of interventions in locations where donations are non-remunerated, allowing us to explore the heterogeneity of results. We will also explore potential differences over time (i.e., by year of publication) and between studies investigating convalescent vs. source plasma. We will also conduct subgroup analyses for equity-deserving groups of interest, such as gbMSM and racialized donors. Depending on the number of results, a sensitivity analysis will be conducted based on risk of bias (low, medium, high).

#### Unit-of-analysis concerns

For cluster-randomized trials, unit of analysis issues are possible if researchers do not account for the effect of clustering on responses [64]. Therefore, to avoid artificially inflating the effects of a cluster-randomized trial, analyses should be conducted at the level of allocation (i.e., clusters, not individuals). For included studies that take clustering into account in their analyses, no adjustments will be made. However, for those studies that do not account for clustering, we will apply a post-hoc correction using an estimated intracluster correlation coefficient (ICC) derived from a comparable literature [65].

#### Sensitivity analyses

To assess the robustness of our results, we will conduct sensitivity analyses by removing (a) any studies that have a sample size an order of magnitude larger than others to inspect if it dominated the results, and (b) any studies judged to be at high risk of bias. We will conduct other sensitivity analyses as necessary after viewing any irregularities among the included studies to inspect if they are affecting results [66].

#### Assessing certainty of evidence

To confidently make evidence-based recommendations for behaviour change researchers and blood product collection organizations, the quality of evidence must be judged. We will assess the confidence with which we can say the effect estimates are correct per outcome using the Grading of Recommendations Assessment, Development, and Evaluation (GRADE) framework [67]. The GRADE evidence rating system provides rating criteria that allow research teams to transparently judge studies in five domains (risk of bias, inconsistency, indirectness, imprecision, and publication bias) and classifies evidence into one of four categories of quality (high, moderate, low, or very low). A Summary of Findings (SoF) table will be produced using GRADEPro software [67] to show the results of the GRADE assessment and the justifications for decisions made.

#### Potential amendments to analysis plan

Should there not be a sufficient number of studies to perform our planned analyses, we may elect to combine the included studies into one review and conduct descriptive analyses only.

## Discussion

Plasma donation plays a vital role in public health preparedness and improving population health, yet the current donation system in many countries, such as Canada, is not sustainable.

Our planned reviews will provide a nuanced understanding of important intervention components to consider in the design of optimal recruitment and retention strategies, including multi-level barriers and enablers, relevant behaviour-change techniques, and the potential need to tailor interventions to specific populations and cultural contexts.

## Supporting information

S1 Appendix PRISMA-P checklist. This is the S1 Appendix legend (legend optional).
(PDF)

S2 Appendix Draft MEDLINE search strategy. This is the S2 Appendix legend (legend optional).
(PDF)
