# Supplementary material for: Barriers and enablers to and strategies for promoting domestic plasma donation throughout the world: Overarching protocol for three systematic reviews
Source: PLoS One. 2023 Dec 21;18(12):e0296104. doi: 10.1371/journal.pone.0296104 (PMC10735017; doi:10.1371/journal.pone.0296104)
Supplement: S2 Appendix — This is the S2 Appendix legend (legend optional). (PDF) [file pone.0296104.s002.pdf]

S2. Draft MEDLINE Search Strategy

Ovid MEDLINE(R) ALL <1946 to July 03, 2023>

1	exp plasma/	31442
2	Plasmapheresis/	9195
3	plasma*.tw,kf.	1033701
4	or/1-3	1040882
5	Blood Donors/	22002
6	((blood or plasma*) adj5 (donor* or donation*)).tw,kf.	44363
7	(plasma* adj2 collection).tw,kf.	669
8	or/5-7	52604
9	4 and 8	9513
10	(enabler* or barrier* or obstacle* or hurdle* or deterrent*).tw,kf.	483598
11	motivation/ or intention/	94143
12	Health Knowledge, Attitudes, Practice/	126573
13	Altruism/	7746
14	(motiv* or intent* or altruis*).tw,kf.	342356
15	(incentive* or disincentive*).tw,kf.	37503
16	((donor* or donation*) adj3 (attitude* or Belief* or determinant* or experience* or factor* or feeling* or knowledge or opinion* or perceive*or perception* or view*)).tw,kf.	6871
17	"surveys and questionnaires"/ or health care surveys/ or health surveys/	643913
18	(survey* or questionnaire* or interview*).tw,kf.	1671983
19	Health Promotion/	81362
20	promotion.tw,kf.	124858
21	health promot*.tw,kf.	53253
22	(promot* adj3 (strateg* or intervention*)).tw,kf.	33113
23	or/10-22	2717165
24	9 and 23	693
25	exp animals/ not humans/	5135938
26	24 not 25	679
